# Supplementary material for: A photoswitchable inhibitor of TREK channels controls pain in wild-type intact freely moving animals
Source: Nat Commun. 2023 Mar 1;14:1160. doi: 10.1038/s41467-023-36806-4 (PMC9977718; doi:10.1038/s41467-023-36806-4)
Supplement: Supplementary file 1 — Supplementary Information [file 41467_2023_36806_MOESM1_ESM.pdf]

Supplementary Information

For

A photoswitchable inhibitor of TREK channels controls pain in wild-type intact freely moving animals

Arnaud Landra-Willm[1,2,3], Ameya Karapurkar[4], Alexia Duveau[5], Anne Amandine Chassot[1,2,3], Lucille Esnault[6], Gerard Callejo[7,8], Marion Bied[1,2,3], Stephanie Häfner[1,2,3], Florian Lesage[2,3,6], Brigitte Wdziekonski[1,2,3], Anne Baron[2,3,6], Pascal Fossat[5], Laurent Marsollier[6], Xavier Gasull[7,8], Eric Boué-Grabot[4], Michael A. Kienzler[9#*] and Guillaume Sandoz[1,2,3#*]

Affiliations :

[1]Université Côte d'Azur, CNRS, INSERM, iBV, France

[2]Laboratories of Excellence, Ion Channel Science and Therapeutics, Nice, France

[3]Fédération Hospitalo-Universitaire InovPain, Cote d'Azur University, University Hospital
13 Centre Nice, Nice, Provence-Alpes-Côte d'Azur, France

[4]University of Maine Department of Chemistry, 178 Munson Rd. Orono, ME 04473, USA

[5]Univ. Bordeaux, CNRS, IMN, UMR 5293, F-33000 Bordeaux, France

[6]Equipe ATOMycA, U1232 Centre de Recherche en Cancérologie et Immunologie Nantes-Angers, Institut National de la Santé et de la Recherche Médicale (INSERM), Université de Nantes, Université d'Angers, Angers, France

[6]Centre National de la Recherche Scientifique, Institut de Pharmacologie Moléculaire et Cellulaire, Labex ICST, Université Côte d'Azur, INSERM, Valbonne, France.

[7]Neurophysiology Laboratory, Department of Biomedicine, Medical School, Institute of Neurosciences, Universitat de Barcelona, c. Casanova 143, 08036, Barcelona, Spain

[8]Institut d'Investigacions Biomediques August Pi i Sunyer (IDIBAPS). c. Villarroel 170, 08036 Barcelona, Spain

[9]University of Connecticut Department of Chemistry, 55 N. Eagleville Rd Storrs, CT 06269, USA

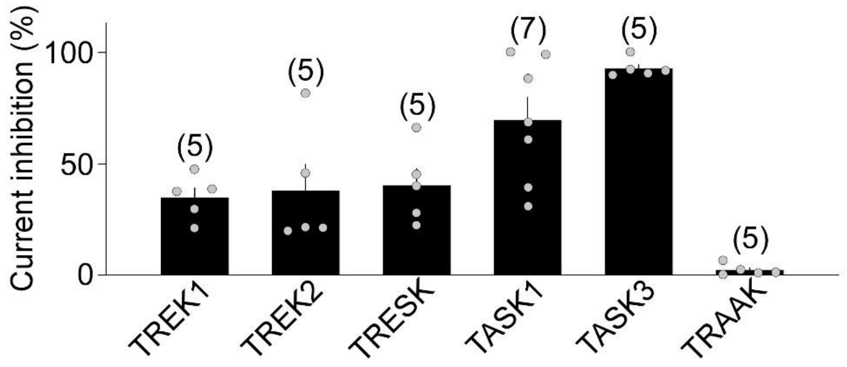

Supplementary Figure 1. ML365 is an antagonist of TREK1/2 and TRESK.

Bar graph summarizing the current inhibition (%) of TREK1, TREK2, TRESK, TASK1, TASK3 and TRAAK at -60 mV by 5µM of ML365. For each channel, n was obtained from one experiment. Data are represented as mean ± SEM. The numbers of tested cells are indicated in parentheses on the graph.

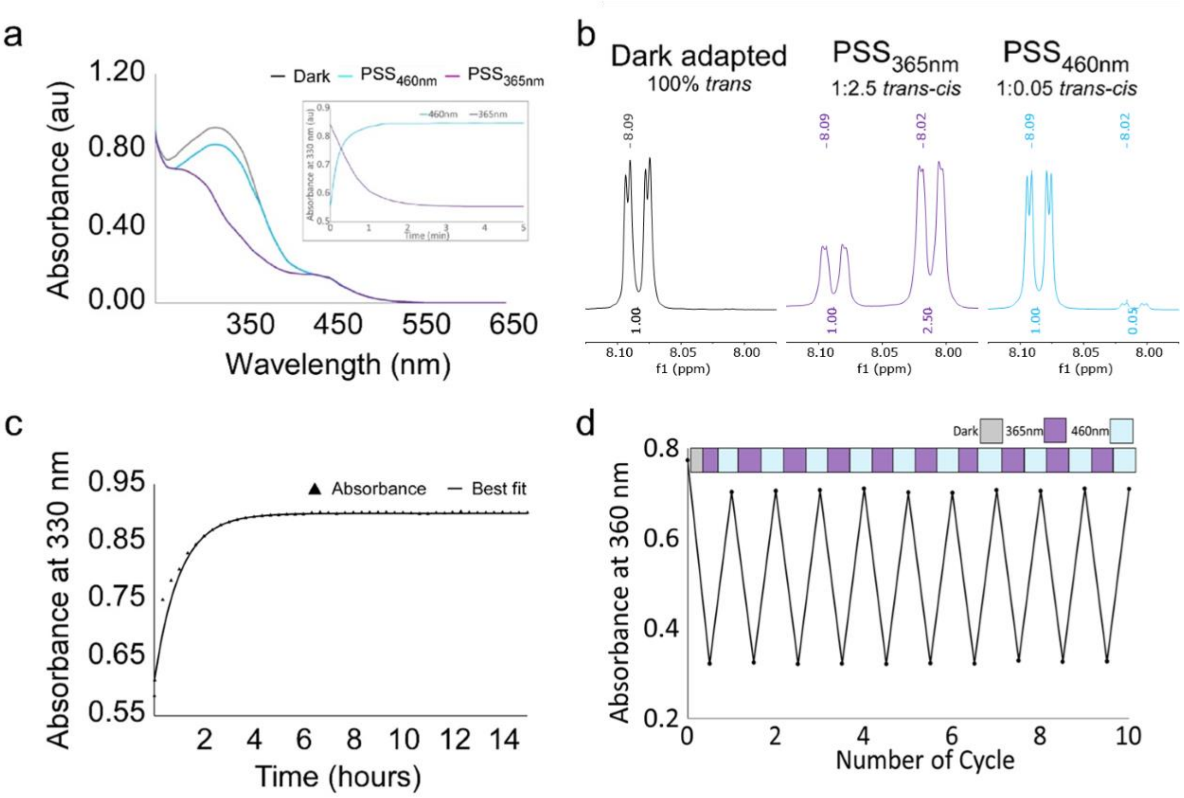

Supplementary Figure 2. Photochemical characterization of LAKI.

(a) UV-Visible absorbance spectra of LAKI (5 µM in HEPES solution containing 0.1% DMSO) before and after photoirradiation (blue 460 nm light and UV 365 nm light). Inset shows the rate of switching between blue 460 nm light photostationary state and UV 365 nm light photostationary state. (b) ^{1}H-NMR spectra of LAKI collected in CD_3CN showing the photostationary state. (c) Thermal half-life of LAKI (5 µM in HEPES solution containing 0.1% DMSO) observed at 330 nm at 21.5°C to yield a half-life of 0.7 hours (k = 0.99 h^{-1}). (d) Photo-cycling of LAKI observed at 360 nm with alternate irradiation of blue 460 nm light and UV 365 nm light for 5 min each.

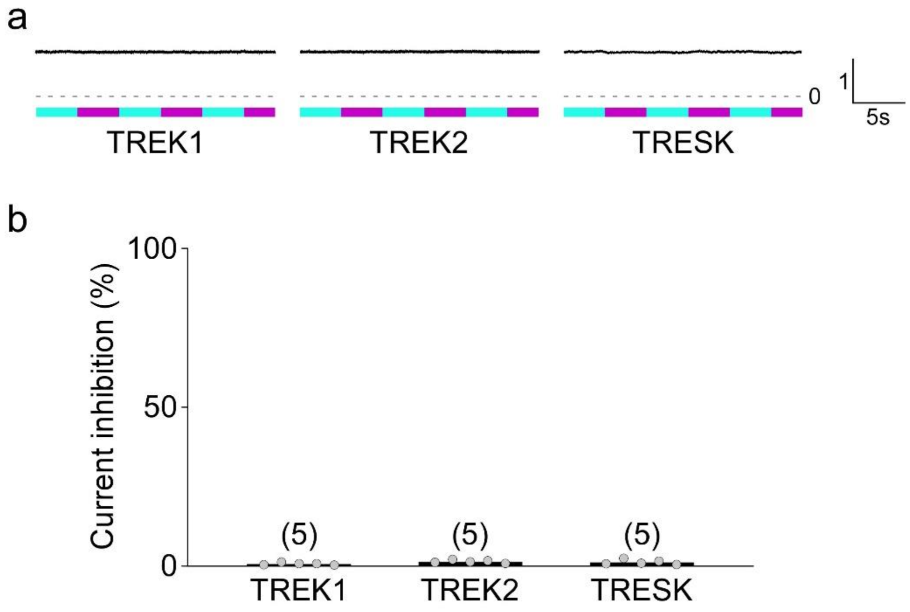

Supplementary Figure 3. LAKI does not act intracellularly.

Normalized whole-cell current recordings elicited at 0 mV from HEK293T cells expressing TREK1, TREK2 and TRESK in the presence of intracellular LAKI (5 µM) upon alternating illumination at 480 nm (blue) and 365 nm (magenta). (b) Bar graph summarizing the current inhibition (%) of TREK1, TREK2 and TRESK at 0 mV. For each channel, n was obtained from one experiment. Statistical significance was determined by QuasiBinomial GLM followed by Tukey's post-test (non-significant $p > 0.05$). Data are represented as mean ± SEM. The numbers of tested cells are indicated in parentheses on the graph.

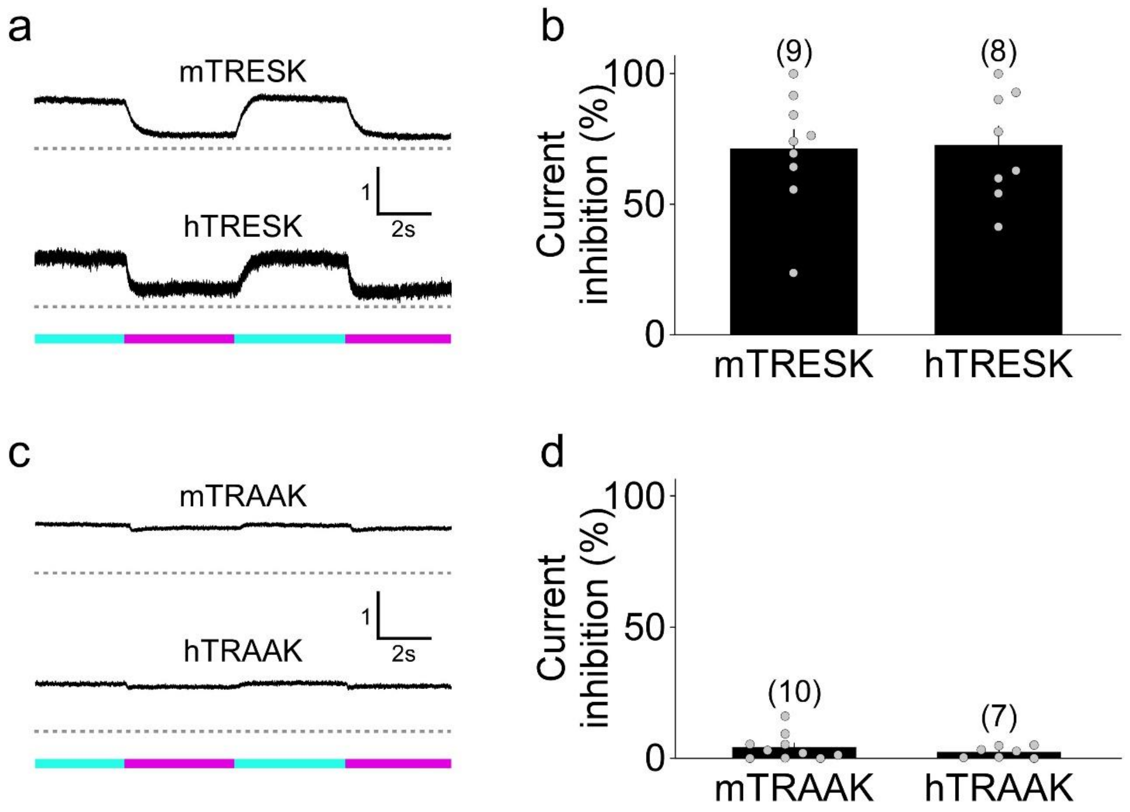

Supplementary Figure 4. LAKI is similarly efficient to photoblock TRESK and TRAAK channels from different species.

(a) Normalized whole-cell current recording elicited at -60 mV from HEK293T cells expressing either mouse or human TRESK in the presence of LAKI (5 µM) upon alternating illumination at 480 nm (blue) and 365 nm (magenta). (b) Bar graph summarizing the current inhibition (%) of mouse and human TRESK at -60 mV. n was obtained from two independent experiments. (c) Normalized whole-cell current recording elicited at -60 mV from HEK293T cells expressing either mouse or human TRAAK in the presence of LAKI (5 µM) upon alternating illumination at 480 nm (blue) and 365 nm (magenta). (d) Bar graph summarizing the current inhibition (%) of mouse and human TRAAK at -60 mV. n was obtained from one experiment. Statistical significance was determined by QuasiBinomial GLM (non significant $p > 0.05$). Data are represented as mean ± SEM. The numbers of tested cells are indicated in parentheses on the graph.

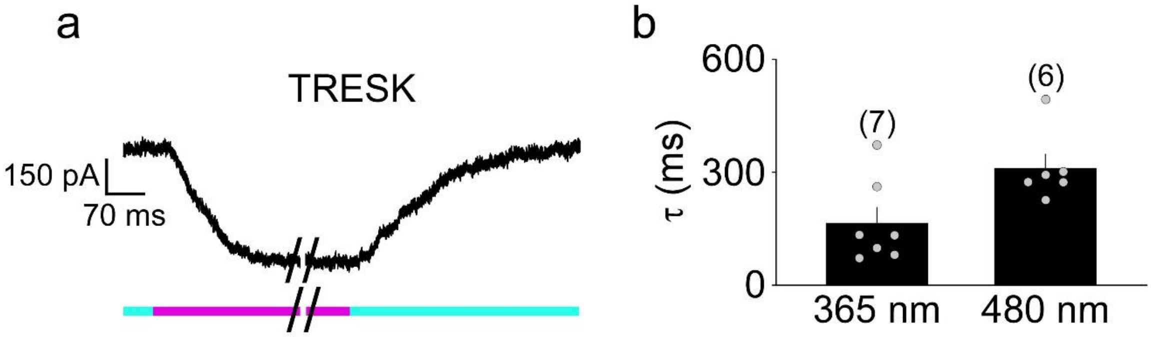

Supplementary Figure 5. LAKI blocking and unblocking kinetics on TRESK channel.

(a) Whole-cell current recordings elicited at 0 mV from HEK293T cells expressing TRESK in presence of LAKI (5 µM) upon alternating illumination at 480 nm (blue) and 365 nm (magenta). (b) Bar graph summarizing the LAKI blocking (365 nm) and unblocking (480 nm) time constants tau on TRESK channel at 0 mV. n was obtained from one experiment. Data are represented as mean ± SEM. The numbers of tested cells are indicated in parentheses on the graph.

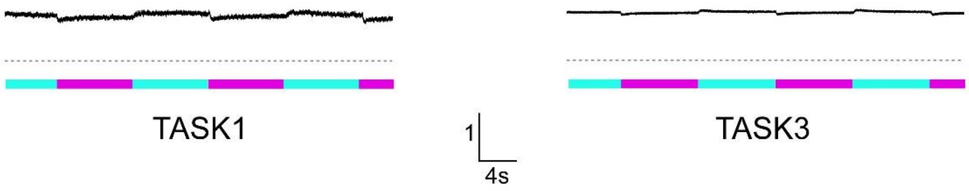

Supplementary Figure 6. LAKI does not endow light sensitivity to TASK1 and TASK3 channels.

Normalized whole-cell current recordings elicited at -60 mV from HEK293T cells expressing TASK1 or TASK3 in the presence of LAKI (5 µM) upon alternating illumination at 480 nm (blue) and 365 nm (magenta).

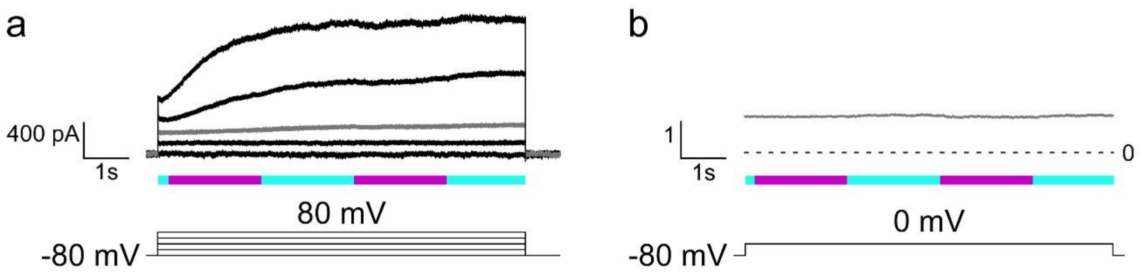

Supplementary Figure 7. LAKI does not photocontrol KCNQ1-KCNE1.

Whole-cell current recordings elicited by voltage-steps (from -80 to 80 mV, 20 mV increments, 8 s in duration) from HEK293T cells co-expressing KCNQ1 and KCNE1 in the presence of LAKI (5 µM) upon alternating illumination at 480 nm (blue) and 365 nm (magenta) (Current elicited at 0 mV represented by gray trace). (b) Zoom-in of the normalized whole-cell current recording under LAKI photomodulation at 0 mV.

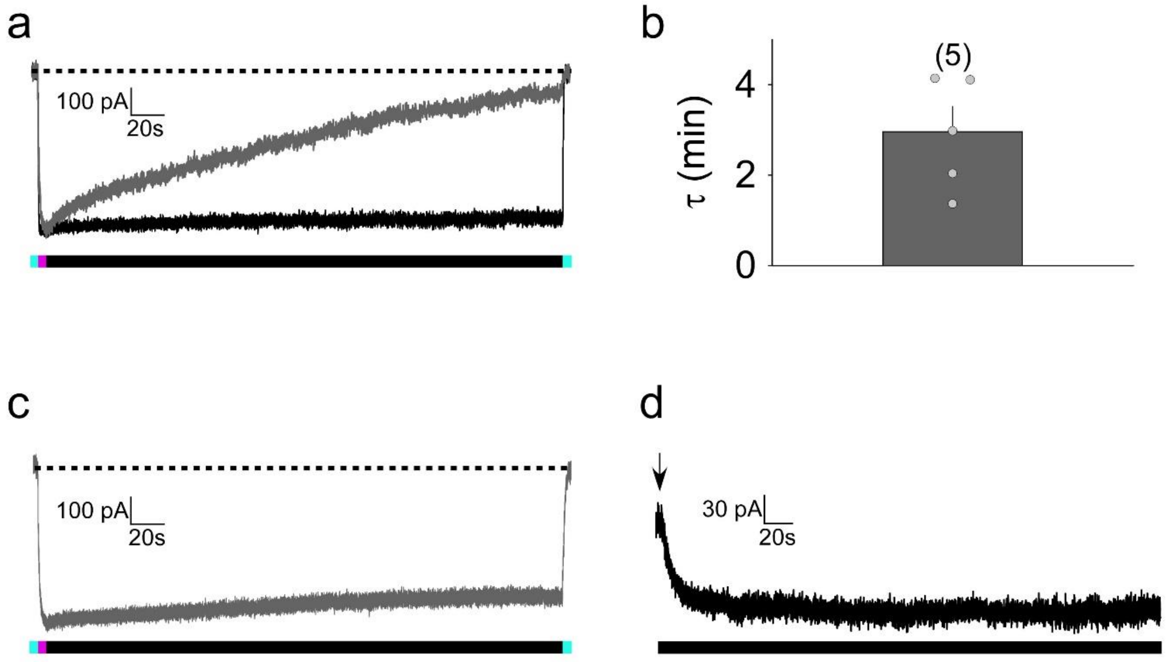

Supplementary Figure 8. LAKI functional bistability is dependent of UV light intensity and concentration. (a) Representative whole-cell current recordings elicited at -60 mV from HEK293T expressing TRESK in the presence of LAKI (10 µM) upon illumination at 480 nm (blue) and 365 nm (magenta) or in the dark. Dark and grey traces represent cell illuminated respectively with 4.32 and 1.05 $mW.cm^{-2}$ UV light. (b) Bar graph showing the time constant τ of LAKI relaxation on TRESK channel after 1.05 $mW.cm^{-2}$ illumination at 365 nm. n was obtained from one experiment. (c) Representative whole-cell current recordings elicited at -60 mV from HEK293T expressing TRESK in the presence of LAKI (100 µM) upon illumination at 480 nm (blue) and 365 nm (magenta) or in the dark. (d) Representative whole-cell current recordings elicited at -60 mV from HEK293T expressing TRESK perfused with *cis*-LAKI (10 µM) maintained at 37°C. Black arrow represents *cis*-LAKI perfusion. Data are represented as mean ± SEM. The numbers of tested cells are indicated in parentheses on the graph.

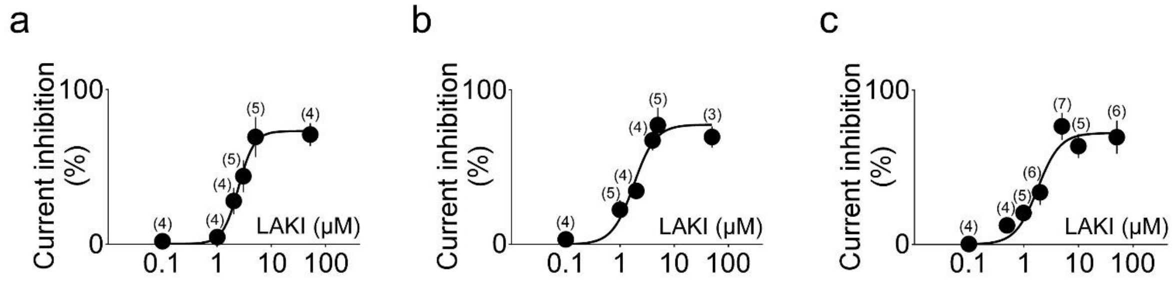

Supplementary Figure 9. LAKI concentration-response curves on TREK1, TREK2 and TRESK channel.

Normalized concentration-response curve of LAKI on whole-cell current elicited at -60 mV from HEK293T cells expressing either (a) TREK1, (b) TREK2 or (c) TRESK. For each channel, n was obtained from one experiment. Data points were fitted using a four-parameter logistic curve. Data are represented as mean ± SEM. The numbers of tested cells are indicated in parentheses above curves.

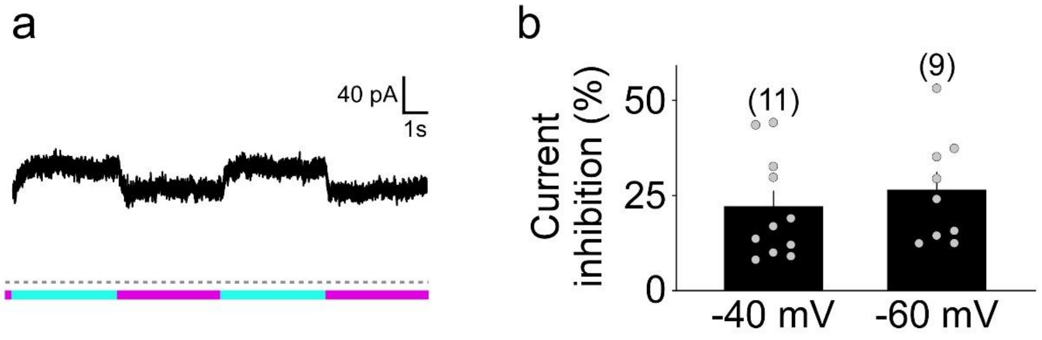

Supplementary Figure 10. TREK channels contribution to neuronal leak current.

(a) Whole-cell current recording elicited at -40 mV from WT TG neurons in presence of LAKI (10 µM) upon alternating illumination at 480 nm (blue) and 365 nm (magenta). (b) Bar graph summarizing the contribution of TREK1/2 and TRESK to WT TG neuron leak current at -40 and -60 mV. n was obtained from 6 mice from two independent experiments. Data are represented as mean ± SEM. The numbers of tested neurons are indicated in parentheses on the graph.

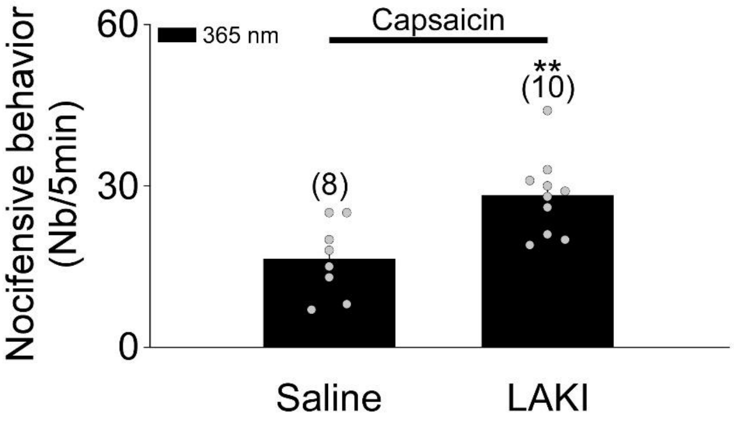

Supplementary Figure 11. LAKI sensitizes the capsaicin pain in rodent.

Bar graph summarizing the average of nocifensive behavior elicited by mice after ocular application of either saline or LAKI (100 µM, 5 µL) solution in presence of Capsaicin (100 µM) after 20s illumination at 365 nm (magenta). n was obtained from two independent experiments. Statistical significance was determined by QuasiPoisson GLM (** $p = 0.00443$).

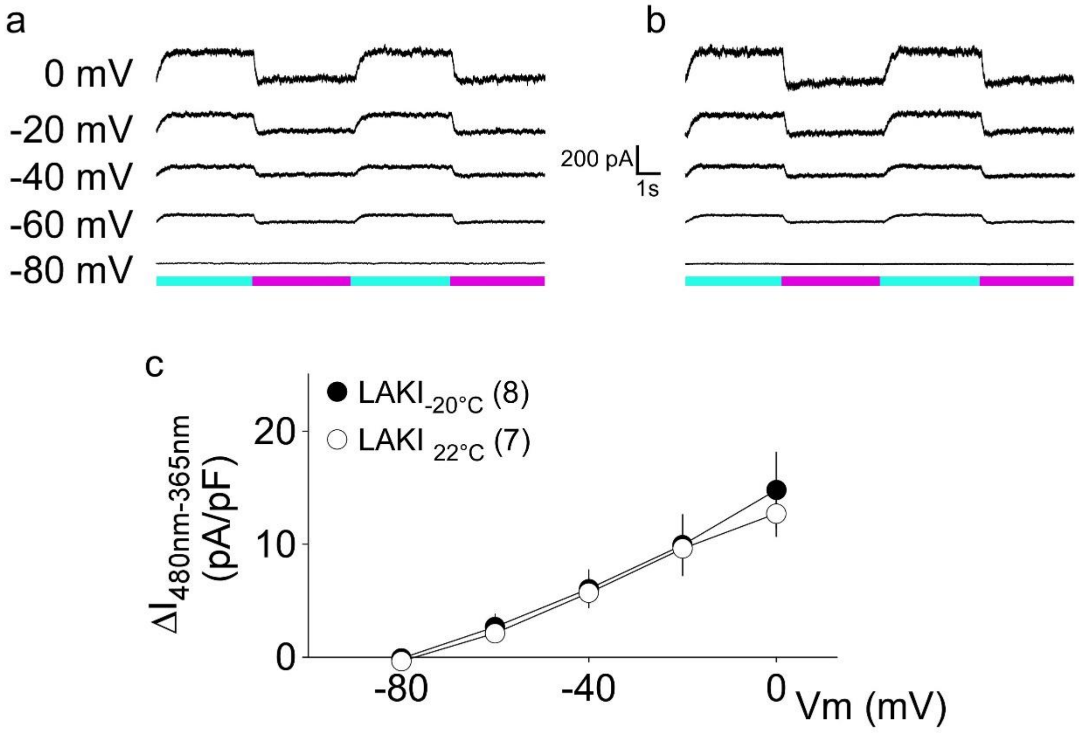

Supplementary Figure 12. LAKI is stable at room temperature for several days.

Whole-cell current recordings elicited at different holding potentials from HEK293T cells expressing TRESK in presence of LAKI (5 µM) stored in dark either (a) at -20°C or (b) at room temperature (≈ 22°C) for 8 days, upon alternating illumination at 480 nm (blue) and 365 nm (magenta). (c) IV relationship of the photocurrent density induced by alternating illumination ($I_{480\ nm} - I_{365\ nm}$) for different holding potentials in HEK293T cells expressing TRESK in presence of LAKI (5 µM) stored in dark either at -20°C or at room temperature (≈ 22°C) for 8 days. n was obtained from one experiment. Data are represented as mean ± SEM.

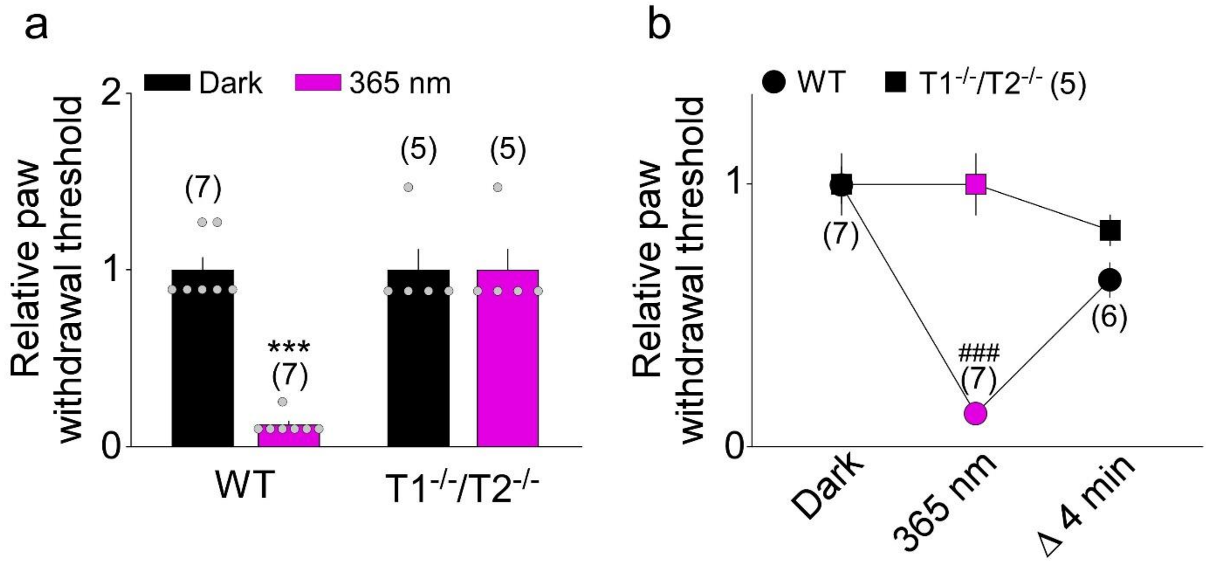

Supplementary Figure 13. LAKI controls mechanical allodynia through the inhibition of TREK channels.

(a) Bar graph summarizing the relative paw withdrawal threshold of either WT or *Trek1*$^{-/-}$*/Trek2*$^{-/-}$ double KO mice injected with LAKI (100 µM, 15 µL) in the dark or after 20s illumination at 365 nm (magenta). n was obtained from one experiment. Statistical significance was determined by Mixed-effects model with repeated measures followed by Holm-Sidak's post-test (*** $p < 0.001$). (b) Graph summarizing the average of the paw withdrawal threshold of WT and double KO mice injected with LAKI (100 µM, 15 µL) relatively to mice injected with saline solution, in the dark or after 20s illumination at 365 nm (magenta). n was obtained from one experiment. Statistical significance between WT and *Trek1*$^{-/-}$*/Trek2*$^{-/-}$ mice in the LAKI-induced sensitization was determined by Mixed-effects model with repeated measures followed by Holm-Sidak's post-test ($^{###}$ $p < 0.001$). Data are represented as mean ± SEM. The numbers of mice are indicated in parentheses on the graph.

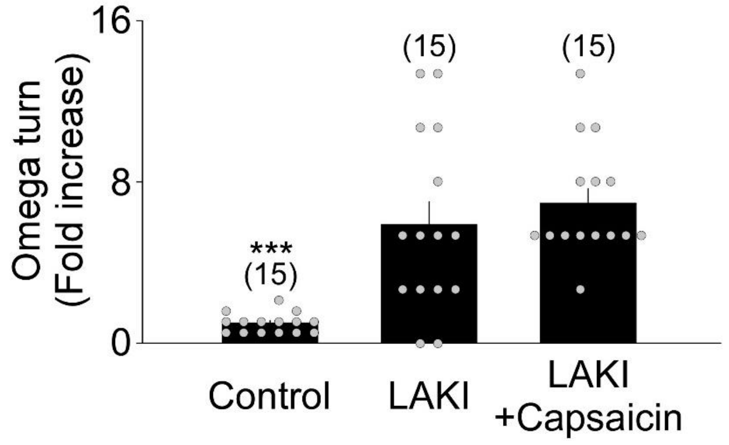

Supplementary Figure 14. LAKI effect on *C. elegans* is not modified by capsaicin.

Bar graph summarizing the fold increase generation of Omega turns made either in presence of LAKI (100 µM), LAKI (100 µM) plus Capsaicin (100 µM) or equivalent DMSO upon 15s illumination at 365 nm. n was obtained from one experiment. Statistical significance was determined by QuasiPoisson GLM followed by Dunnett's post-test (*** $p < 0.001$ versus LAKI). Data are represented as mean ± SEM adjusted for propagation of uncertainties. The numbers of *C. elegans* are indicated in parentheses on the graph.

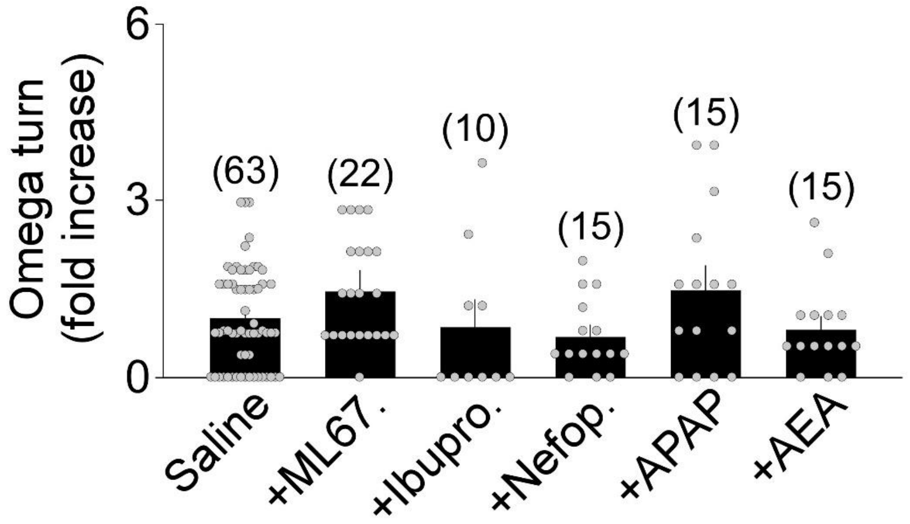

Supplementary Figure 15. Analgesics did not reduce endogenous worm sensitivity to UV light.

Bar graph summarizing the relative average of Omega turns made either in presence of ML67.33 (80 µM), Ibuprofen (100 µM), Nefopam (100 µM), APAP (100 µM), AEA (100 µM) or equivalent DMSO upon 15s illumination at 365 nm. n for Saline was obtained from four independent experiments, n for analgesic molecules was obtained from one experiment. Statistical significance was determined by QuasiPoisson GLM followed by Dunnett's post-test (non significant $p > 0.05$ versus control). Data are represented as mean ± SEM adjusted for propagation of uncertainties. The numbers of *C. elegans* are indicated in parentheses on the graph.

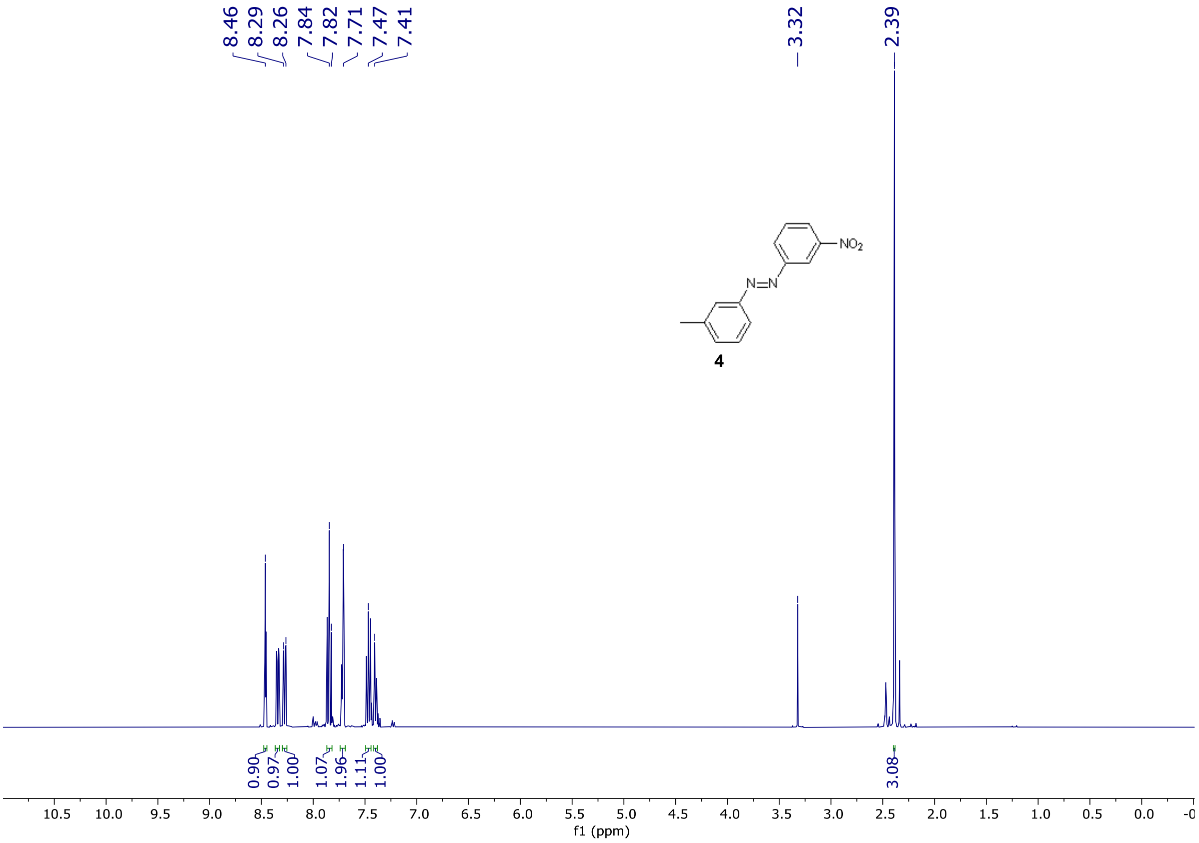

Supplementary Figure 16. **^{1}H NMR spectrum of compound 4.**

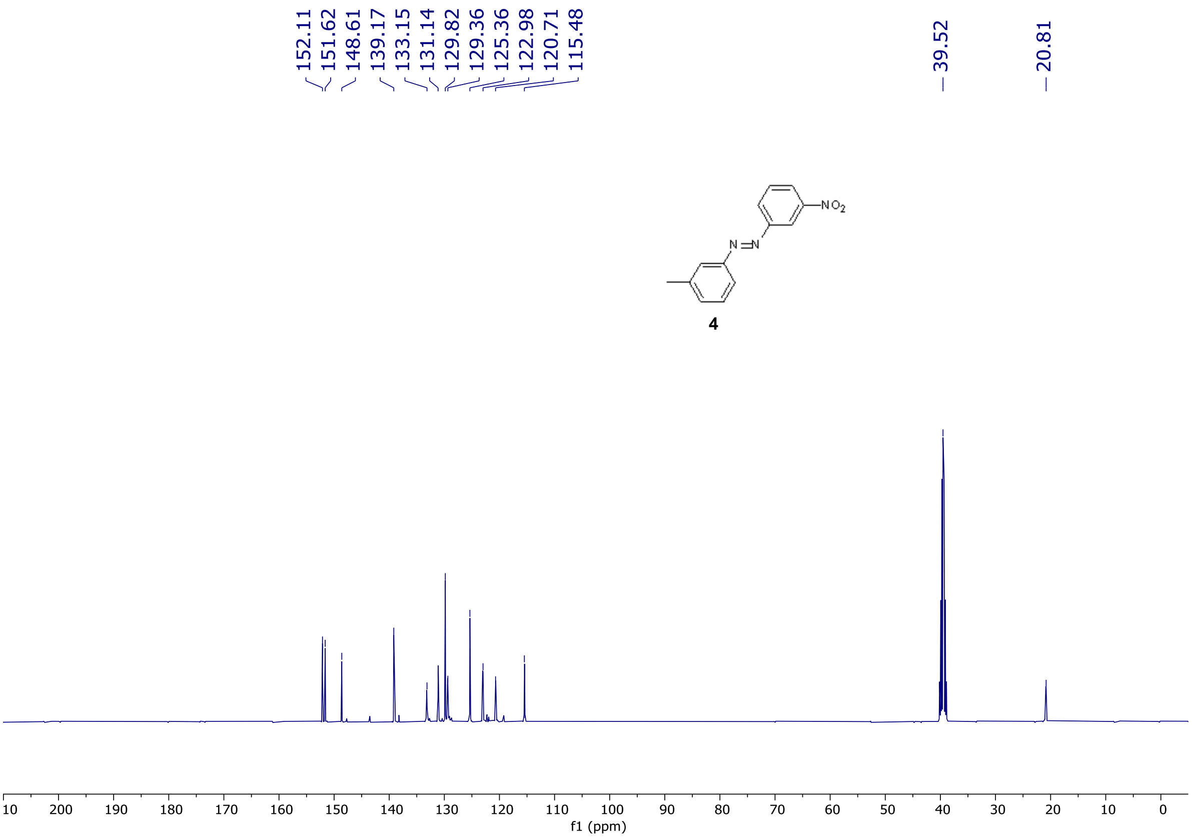

Supplementary Figure 17. **^{13}C NMR spectrum of compound 4.**

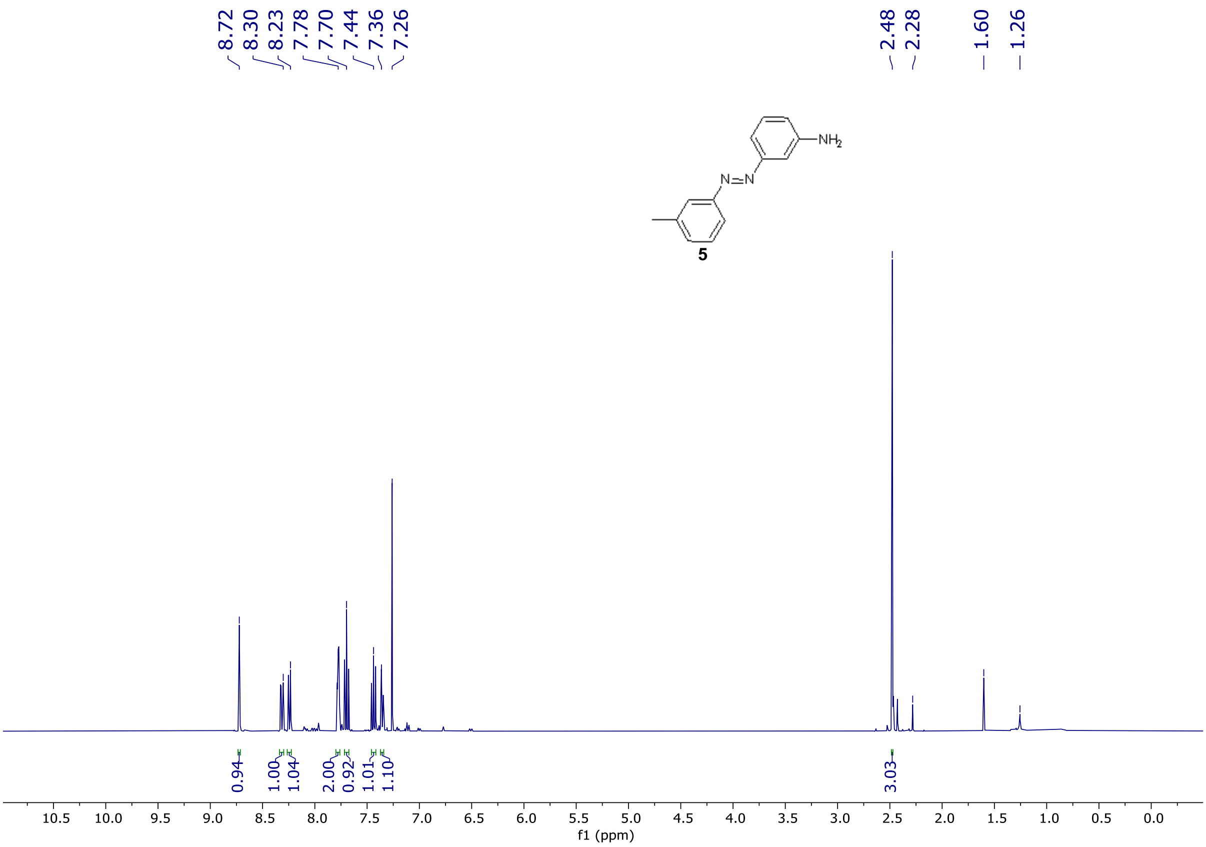

Supplementary Figure 18. ^{1}H NMR spectrum of compound 5.

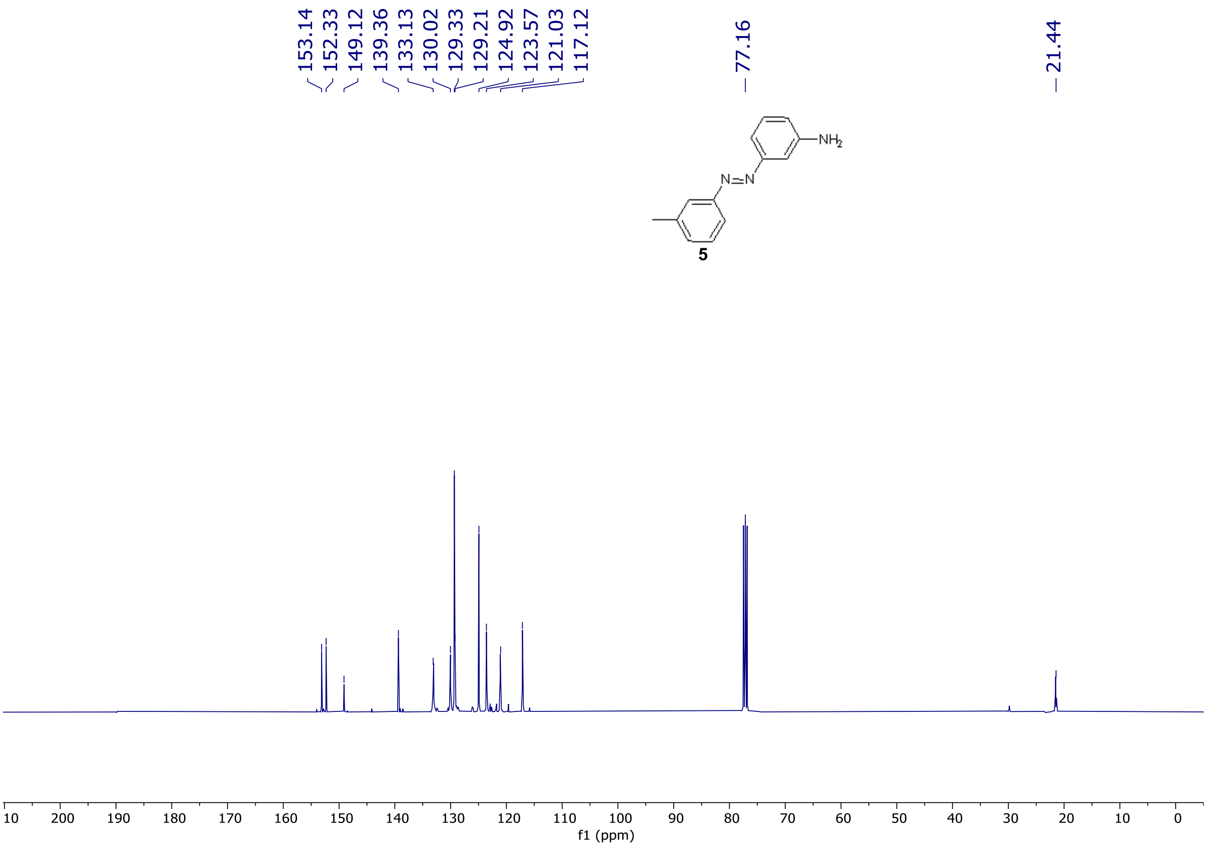

Supplementary Figure 19. ^{13}C NMR spectrum of compound 5.

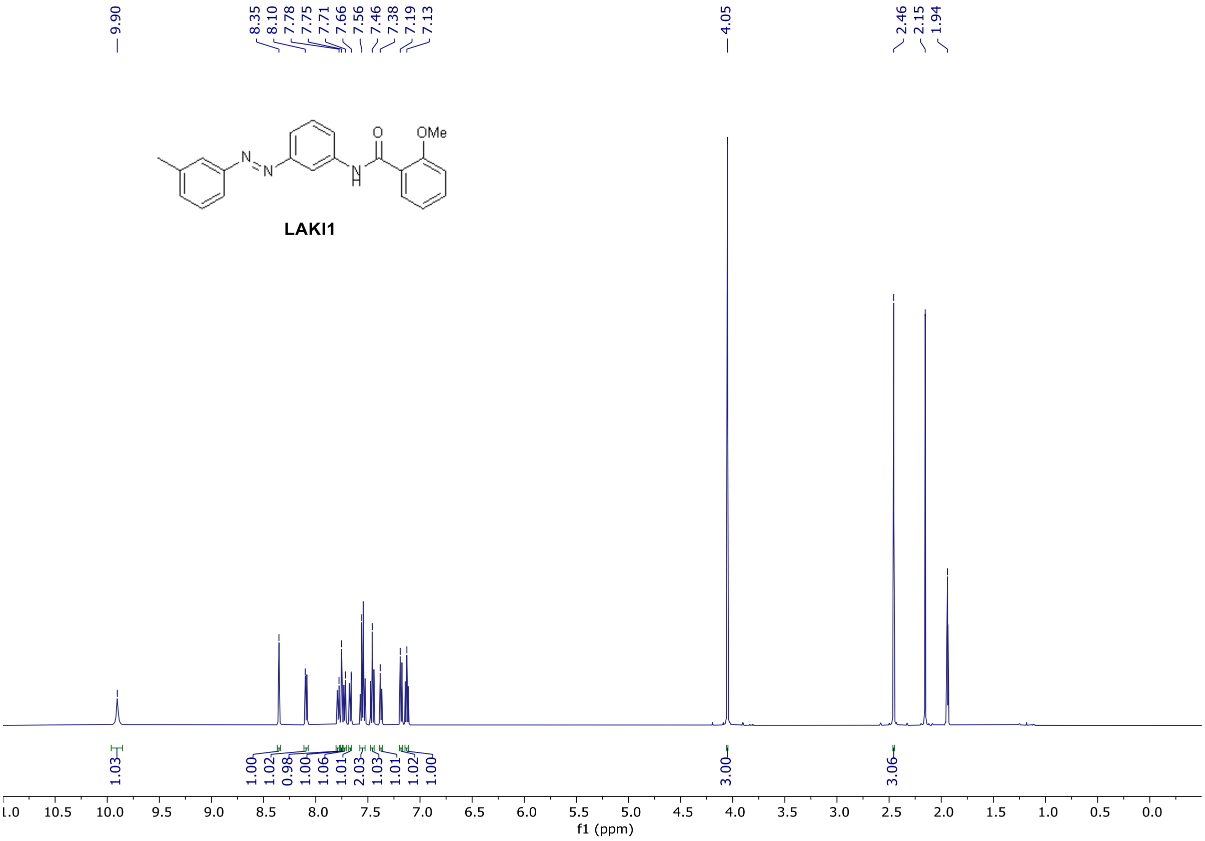

Supplementary Figure 20. ^{1}H NMR spectrum of compound LAKI.

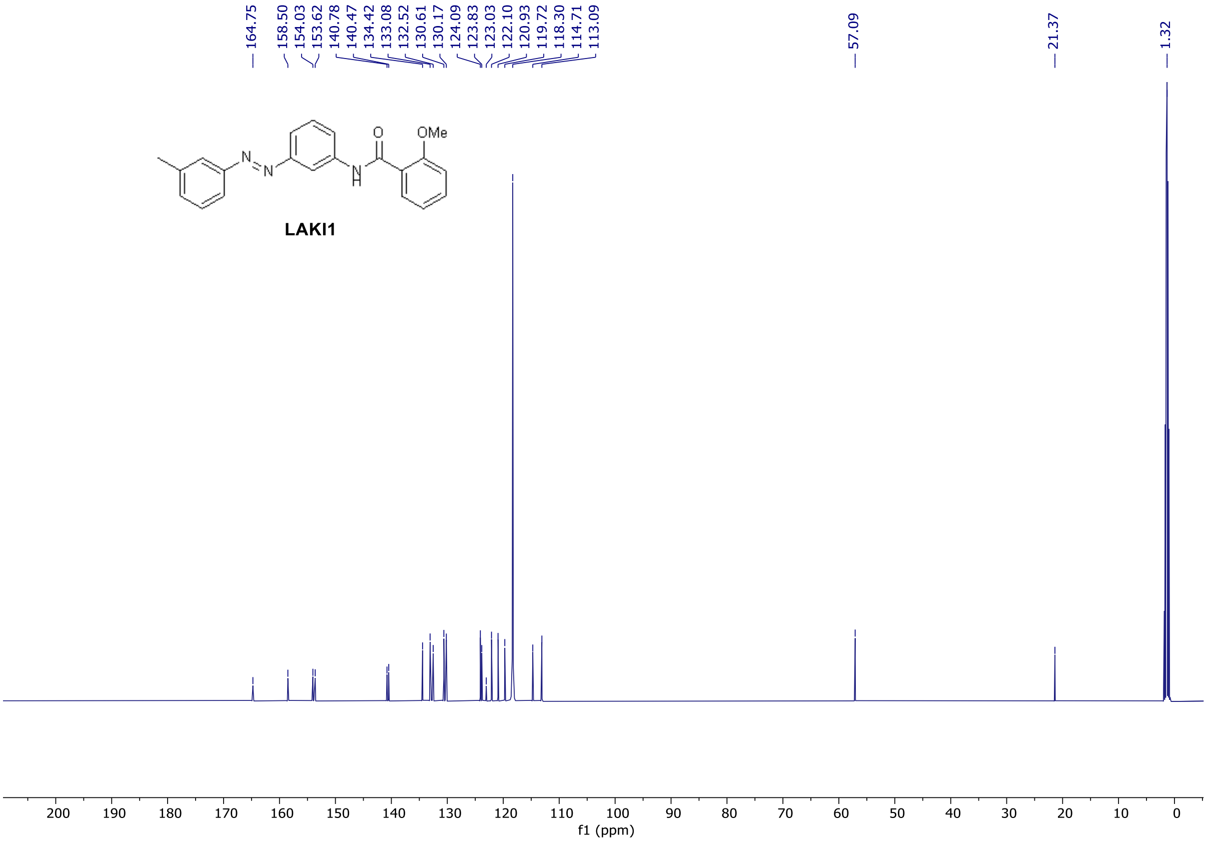

Supplementary Figure 21. ^{13}C NMR spectrum of compound LAKI.

Supplementary statistical results

Supplementary table 1. Statistical results for Figure 1.

	**Statistical analysis**		**Result**
(c)	QuasiBinomial GLM	Expressed channel	$\chi^2(5,27)$; p<0.001

Supplementary table 2. Statistical results for Figure 2.

	**Statistical analysis**		**Result**
(d)	Repeated Measures Mixed-effects model	Condition	F(2,25)=49.78 ; p<0.001
		HMP	F(1.941,45.13)=85.53 ; p<0.001
		Condition*HMP	F(8,93)=33.75 ; p<0.001
(f)	Paired t-test	Wavelength	t(8)=3.788 ; p = 0.0053

HMP = Holding Membrane Potential

Supplementary table 3. Statistical results for Figure 3.

	**Statistical analysis**		**Result**
(c)	Poisson GLM	Illumination/Saline	z(1,13) ; p>0.99
		Illumination/LAKI	z(1,19) ; p<0.001
(d) Scratching	Poisson GLM	Illumination/Saline	z(1,13) ; p>0.99
		Illumination/LAKI	z(1,19) ; p=0.00282
(d) Wiping	Poisson GLM	Illumination/Saline	z(1,13) ; p>0.83
		Illumination/LAKI	z(1,19) ; p=0.00175
(e)	Repeated Measures Two-way ANOVA	Treatment	F(1,12)=1.97 ; p=0.1858
		Illumination	F(1,12)=30.35 ; p<0.001
		Treatment*Illumination	F(1,12)=7.416 ; p=0.0185
(f)	Repeated Measures Mixed-effects model	Treatment	F(1,12)=6.603 ; p=0.0246
		Illumination	F(3,25)=6.701 ; p=0.0018
		Treatment*Illumination	F(3,25)=6.653 ; p=0.0019
(g)	Repeated Measures Mixed-effects model	Treatment	F(1,11)=1.157 ; p=0.3050
		Illumination	F(1,11)=12.39 ; p=0.0048
		Treatment*Illumination	F(1,11)=2.575 ; p=0.1369
(h)	Repeated Measures Mixed-effects model	Day	F(1.756,7.025)=2.48 ; p=0.1552
		Wavelength	F(1,4)=25.83 ; p=0.0071
		Day*Wavelength	F(1.259,4.196)=12.86 ; p=0.0194

Supplementary table 4. Statistical results for Figure 4.

	Statistical analysis		Result
(a)	Repeated Measures Mixed-effects model	Treatment	F(1,6)=33.55 ; p=0.0012
		Illumination	F(1,6)=222.5 ; p<0.001
		Treatment*Illumination	F(1,6)=222.5 ; p<0.001
(b)	Repeated Measures Mixed-effects model	Illumination	F(4,21)=119.7 ; p<0.001

Supplementary table 5. Statistical results for Figure 5.

	Statistical analysis		Result
(c)	QuasiPoisson GLM	Treatment/Dark	t(1,131) ; p>0.99
		Treatment/365 nm	t(1,132) ; p<0.001
		Treatment/480 nm	t(1,126) ; p=0.118
(d)	QuasiPoisson GLM	Treatment	χ^2(6,209) ; p<0.001

Supplementary table 6. Statistical results for Supplementary Figure 3.

	Statistical analysis		Result
(b)	QuasiBinomial GLM	Expressed channel	χ^2(2,12) ; p=0.1071

Supplementary table 7. Statistical results for Supplementary Figure 4.

	Statistical analysis		Result
(b)	QuasiBinomial GLM	Species	t(1,15) ; p=0.9001
(d)	QuasiBinomial GLM	Species	t(1,15) ; p=0.35

Supplementary table 8. Statistical results for Supplementary Figure 9.

	Statistical analysis	Result
(a)	Sigmoidal 4PL	IC_{50}=2.45<br>Hill slope=3.02<br>Degrees of Freedom=23<br>R^2=0.737
(b)	Sigmoidal 4PL	IC_{50}=1.79<br>Hill slope=2.17<br>Degrees of Freedom=22<br>R^2=0.779
(c)	Sigmoidal 4PL	IC_{50}=1.75<br>Hill slope=2.13<br>Degrees of Freedom=34<br>R^2=0.701

Supplementary table 9. Statistical results for Supplementary Figure 11.

	Statistical analysis		Result
	QuasiPoisson GLM	Treatment	t(1,16) ; p=0.00443

Supplementary table 10. Statistical results for Supplementary Figure 13.

	Statistical analysis		Result
(a)	Repeated Measures Mixed-effects model	Genotype	F(1,10)=30.83 ; p<0.001
		Illumination	F(1,10)=26.77 ; p<0.001
		Genotype*Illumination	F(1,10)=26.77 ; p<0.001
(b)	Repeated Measures Mixed-effects model	Genotype	F(1,10)=31.51 ; p<0.001
		Illumination	F(2,19)=16.93 ; p<0.001
		Genotype*Illumination	F(2,19)=18.38 ; p<0.001

Supplementary table 11. Statistical results for Supplementary Figure 14.

	Statistical analysis		Result
	QuasiPoisson GLM	Treatment	χ^2(2,42) ; p<0.001

Supplementary table 12. Statistical results for Supplementary Figure 15.

	Statistical analysis		Result
	QuasiPoisson GLM	Treatment	$\chi^2(5,134)$; p=0.04957

All the performed statistical tests were two-sided.
