# Supplementary material for: A photoswitchable inhibitor of TREK channels controls pain in wild-type intact freely moving animals
Source: Nat Commun. 2023 Mar 1;14:1160. doi: 10.1038/s41467-023-36806-4 (PMC9977718; doi:10.1038/s41467-023-36806-4)
Supplement: Supplementary file 3 — Reporting Summary [file 41467_2023_36806_MOESM3_ESM.pdf]

nature portfolio

Corresponding author(s): Guillaume Sandoz

Last updated by author(s): Jan 19, 2023

Reporting Summary

Nature Portfolio wishes to improve the reproducibility of the work that we publish. This form provides structure for consistency and transparency in reporting. For further information on Nature Portfolio policies, see our Editorial Policies and the Editorial Policy Checklist.

Statistics

For all statistical analyses, confirm that the following items are present in the figure legend, table legend, main text, or Methods section.

n/a	Confirmed	
☐	☒	The exact sample size (*n*) for each experimental group/condition, given as a discrete number and unit of measurement
☐	☒	A statement on whether measurements were taken from distinct samples or whether the same sample was measured repeatedly
☐	☒	The statistical test(s) used AND whether they are one- or two-sided<br>*Only common tests should be described solely by name; describe more complex techniques in the Methods section.*
☐	☒	A description of all covariates tested
☐	☒	A description of any assumptions or corrections, such as tests of normality and adjustment for multiple comparisons
☐	☒	A full description of the statistical parameters including central tendency (e.g. means) or other basic estimates (e.g. regression coefficient) AND variation (e.g. standard deviation) or associated estimates of uncertainty (e.g. confidence intervals)
☐	☒	For null hypothesis testing, the test statistic (e.g. *F*, *t*, *r*) with confidence intervals, effect sizes, degrees of freedom and *P* value noted<br>*Give P values as exact values whenever suitable.*
☒	☐	For Bayesian analysis, information on the choice of priors and Markov chain Monte Carlo settings
☒	☐	For hierarchical and complex designs, identification of the appropriate level for tests and full reporting of outcomes
☒	☐	Estimates of effect sizes (e.g. Cohen's *d*, Pearson's *r*), indicating how they were calculated

Our web collection on statistics for biologists contains articles on many of the points above.

Software and code

Policy information about availability of computer code

Data collection	pClamp 11.0.3, Micro-Manager 1.4.21, Spike2 8.04
Data analysis	pClamp 11.0.3, SigmaPlot 11.0, GraphPad Prism 8.0.2, Excel, Fiji 2.0.0, Logger Lite 1.9.4

For manuscripts utilizing custom algorithms or software that are central to the research but not yet described in published literature, software must be made available to editors and reviewers. We strongly encourage code deposition in a community repository (e.g. GitHub). See the Nature Portfolio guidelines for submitting code & software for further information.

Data

Policy information about availability of data

All manuscripts must include a data availability statement. This statement should provide the following information, where applicable:

- Accession codes, unique identifiers, or web links for publicly available datasets
- A description of any restrictions on data availability
- For clinical datasets or third party data, please ensure that the statement adheres to our policy

The main data generated in this study are provided in the Source Data file.

March 2021

Human research participants

Policy information about [studies involving human research participants and Sex and Gender in Research.]

Reporting on sex and gender	NA
Population characteristics	NA
Recruitment	NA
Ethics oversight	NA

Note that full information on the approval of the study protocol must also be provided in the manuscript.

Field-specific reporting

Please select the one below that is the best fit for your research. If you are not sure, read the appropriate sections before making your selection.

☒ Life sciences ☐ Behavioural & social sciences ☐ Ecological, evolutionary & environmental sciences

For a reference copy of the document with all sections, see nature.com/documents/nr-reporting-summary-flat.pdf

Life sciences study design

All studies must disclose on these points even when the disclosure is negative.

Sample size	The sample size was based on previous experiments and homogeneity of the raw data. Sample sizes were also based on previous studies : Sandoz et al, 2012 and Royal et al, 2019.
Data exclusions	For electrophysiology, exclusions were done when the recorded currents were too low to quantify them.<br>For in vivo behavior tests, mice were excluded in case of parasitic movements during the tests.
Replication	Experiments were repetead in separate cells and animals, as shown by the "n" value, which was obtained from the same or different groups.<br>Electrophysiology experiments on HEK cells were done one time except for LAKI characterization on mouse and human TRESK where it has been replicated two times ; LAKI characterization on TG neurons had been replicated two times.<br>In vivo thermal sensitivity experiments carried on mice were done one time.<br>In vivo mechanical sensitivity experiments carried on mice (Figure 4) were replicated seven times (we characterized one mouse per day).<br>In vivo mechanical sensitivity experiments carried on WT and double Knockout TREK1/TREK2 mice (Supplementary Figure 13) were done one time.<br>In vivo nocifensive experiments carried on mice were replicated three times, and two times when characterizing LAKI + capsaicin effect.<br>In vivo experiments carried on C. elegans were replicated four times for Saline and LAKI conditions and one time for analgesic drugs conditions.<br>For all replicated experiments, all replications were sucessful.
Randomization	There was no randomization in these experiements since mice were sex- and age-matched for in vivo experiments.<br>Similarly, for in vivo experiments carried out on C. elegans we took care to only include adult hermaphrodite.
Blinding	Data acquisition and analysis were not carried out in blinded manner. Blinding was not possible because of the availability of researchers who carried out the experiments. Moreover, in several experiments the test and control were done on the same cell or animal (before and after LAKI activation).

Reporting for specific materials, systems and methods

We require information from authors about some types of materials, experimental systems and methods used in many studies. Here, indicate whether each material, system or method listed is relevant to your study. If you are not sure if a list item applies to your research, read the appropriate section before selecting a response.

March 2021

Materials & experimental systems

n/a	Involved in the study
☒	☐ Antibodies
☐	☒ Eukaryotic cell lines
☒	☐ Palaeontology and archaeology
☐	☒ Animals and other organisms
☒	☐ Clinical data
☒	☐ Dual use research of concern

Methods

n/a	Involved in the study
☒	☐ ChIP-seq
☒	☐ Flow cytometry
☒	☐ MRI-based neuroimaging

Eukaryotic cell lines

Policy information about cell lines and Sex and Gender in Research

Cell line source(s)	HEK cells 293T obtained from ATCC (CRL-11268) were transfected using calcium phosphate technic to measure current related to the transfected channels.
Authentication	The cell line was regulary used in recent years. The cell line used was not further authenticated.
Mycoplasma contamination	Cells were regularly tested for mycoplasma contamination using the MycoAlert kit from Lonza and we confirm that cells were negative for mycoplasma contamination.
Commonly misidentified lines (See ICLAC register)	None.

Animals and other research organisms

Policy information about studies involving animals; ARRIVE guidelines recommended for reporting animal research, and Sex and Gender in Research

Laboratory animals	Mouse model : male and female C57BL/6J WT and double KO for TREK1 and TREK2 genes (Royal et al, 2019) adults mice between 9-12 weeks were used for behavioral tests while P1-10 were used for primary culture of Trigeminal neurons.<br>C. elegans model : N2, adult hermaphrodite were used.
Wild animals	No wild animals were used in the study.
Reporting on sex	Male and female mice were used in this manuscript. C. elegans model : N2, adult hermaphrodite were used.
Field-collected samples	No field-collected samples were used.
Ethics oversight	All mouse experiments were conducted according to national and international guidelines and have been approved by the ethical committees (Ministère français de la Recherche, de l'Enseignement et de l'Innovation; University of Barcelona, CEEA, Generalitat 568 de Catalunya, #129/21).

Note that full information on the approval of the study protocol must also be provided in the manuscript.
